# A spatiotemporal gene expression and cell atlases of the developing rat ovary

**DOI:** 10.1111/cpr.13516

**Published:** 2023-06-13

**Authors:** Yong Shi, Yanjie Guo, Jiayi Zhou, Guanshen Cui, Jung‐Chien Cheng, Ying Wu, Yong‐Liang Zhao, Lanlan Fang, Xiao Han, Yun‐Gui Yang, Yingpu Sun

**Affiliations:** ^1^ Henan Key Laboratory of Reproduction and Genetics, Center for Reproductive Medicine The First Affiliated Hospital of Zhengzhou University Zhengzhou China; ^2^ Academy of medical sciences Zhengzhou University Zhengzhou China; ^3^ Key Laboratory of Genomic and Precision Medicine, Collaborative Innovation Center of Genetics and Development, College of Future Technology, Beijing Institute of Genomics Chinese Academy of Sciences Beijing China; ^4^ China National Center for Bioinformation Beijing China; ^5^ University of Chinese Academy of Sciences Beijing China; ^6^ Institute of Stem Cell and Regeneration Chinese Academy of Sciences Beijing China

## Abstract

Normal ovarian development is necessary for the production of healthy oocytes. However, the characteristics of oocytes development at different stages and the regulatory relationship between oocytes and somatic cells remain to be fully explained. Here, we combined scRNA‐seq and spatial transcriptomic sequencing to profile the transcriptomic atlas of developing ovarian of the rat. We identified four components from developing granulosa cells including cumulus, primitive, mural, and luteal cells, and constructed their differential transcriptional regulatory networks. Several novel growth signals from oocytes to cumulus cells were identified, such as JAG1‐NOTCH2 and FGF9‐FGFR2. Moreover, we observed three cumulus sequential phases during follicle development determined by the key transcriptional factors in each cumulus phase (*Bckaf1*, *Gata6*, *Cebpb*, etc.), as well as the potential pinpointed roles of macrophages in luteal regression. Altogether, the single‐cell spatial transcriptomic profile of the ovary provides not only a new research dimension for temporal and spatial analysis of ovary development, but also valuable data resources and a research basis for in‐depth excavation of the mechanisms of mammalian ovary development.

## Graphical abstract

Shi et al. deliver the first single‐cell spatiotemporal gene expression profile in rat ovary and construct differential transcriptional regulatory networks of different cell types. Several novel growth signals and regulons were identified from oocytes to cumulus cells, and during cumulus state transition. These findings provided an important data resources and research basis for in‐depth excavation of the mechanisms of mammalian ovary development.

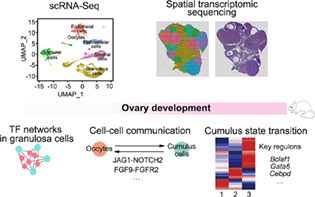

## 1 INTRODUCTION

Ovary is a critical female reproductive organ, serving as the source of oocytes and a major supplier of steroid sex hormones.^1^ It mainly contains cortex, medulla, and follicles with numerous distinct cell types, such as oocytes, granulosa, stromal, endothelial, and immune cells.^2^ Follicles, as the basic functional units of the ovary, are comprised of innermost oocytes surrounded by granulosa cells (GCs) and outer layers of thecal cells (TCs).^3^ Generally, it is distributed throughout the ovarian cortex and subsequently moves into the medulla. The process of follicle development includes: (1) oocyte maturation; (2) GC proliferation and differentiation broadly for supporting the oocyte growth (cumulus GCs) and allowing follicular fluid accumulation in the antrum (mural GCs); (3) TCs are specialized from stromal cells and then surround the follicle, with high vascularization.^4^

Follicle development is dominantly mediated by hormones and key intracellular molecular pathways.^5^ Previous studies have demonstrated that follicle‐stimulating hormone (FSH) receptors are expressed in follicles from primary to later stages, and treatment with FSH and luteinizing hormone (LH) promotes preantral follicle growth.^6^, ^7^ In addition to FSH and LH, some key signalling molecules or pathways are also essential for follicle development. KITL is constitutively expressed by the GCs and interacts with oocytes‐derived KIT to active PI3K‐Akt signalling ensuring the oocytes' growth initiation.^8^, ^9^, ^10^ Meanwhile, GDF9 and BMP15, originating from oocytes, stimulate the proliferation and differentiation of GCs.^11^ CNP encoded by the *NPPC* has recently been found to be a follicle‐stimulating factor. CNP receptors occur in granulosa and cumulus cells of antral and preovulatory follicles.^12^ Treatment of cumulus‐oocyte complexes with CNP stimulates cyclic Guanosine MonoPhosphate (cGMP) production in cumulus cells and inhibits the meiotic resumption of oocytes. All these findings revealed that follicle development is a complicated physiology process involving considerable signalling communications, which remains to be fully explained.

The GC serves as the component of the follicle and the major regulator of oocyte development. A recent study has shown obviously different GC cell populations between aged and young mice by spatial transcriptomic sequencing data,^13^ illustrating the indispensable role of GC cells in maintaining female fertility and follicle cyclic homeostasis. Importantly, abnormal GC function is closely related to reproductive system diseases. For example, *FOXL2* mutation may cause GC tumour formation.^14^ In female monkeys, inactivated anti‐oxidative pathways, increased reactive oxygen species, and apoptosis were observed in GC, suggesting the essential links between GC dysfunction and ovarian aging.^15^ In patients with polycystic ovary syndrome, restricted GC proliferative capacity leads to decreased follicle maturation.^16^ Therefore, deciphering the molecular basis of GC development is of great significance for the diagnosis and treatment of ovarian diseases.

Benefiting from the development of high‐throughput single‐cell sequencing technology, more ovarian cell subtypes have been discovered and the heterogeneity of ovarian cells has been further investigated. In the human ovary, researchers identified five types of ovarian cells including granulosa, stromal, endothelial, immune, and perivascular cells through single‐cell sequencing analysis, and the molecular features of each cell type were presented.^17^ Similarly, another research group has identified four common cell types in mouse ovaries, except for perivascular cells,^18^ and other three extra cell types (germ, erythrocyte, and epithelial cells). Importantly, gene signatures of certain ovarian type between distinct species are different, evidenced by the *Amhr2* specifically exhibited in mouse GCs and *BEX1* extensively expressed in human GCs.^18^, ^19^ Therefore, a comprehensive analysis of molecular characteristics among ovarian cells is still needed. In addition, a previous study has uncovered four subtypes of oocytes at sequential and stepwise developmental stages including primordial, primary, secondary, and antral follicles, and described their gene‐expression changes during the stage‐to‐stage transition of folliculogenesis.^15^ Despite these findings depicting the classification of ovarian cells during the process of follicle development, the detailed molecular mechanisms underlying follicle and GC development, as well as the nature of heterogeneity of ovarian cells are still incomplete. Further elucidation of these critical issues would be highly helpful to our comprehensive understanding of mammalian ovarian development, which is imperatively required in the era of fertility health.

In this study, we used a rat model to establish the first comprehensive single‐cell spatiotemporal transcriptomic landscape of the ovary. We identified six ovarian cell types with distinct gene‐expression signatures characterized by scRNA‐seq and spatial transcriptome (ST). Meanwhile, we distinguished hierarchies of developing GC subtypes and revealed their transcriptional programs underlying for GCs state transformation. We also analysed the transition of cumulus cell functional features before and after ovulation. And finally, the potential role of immune cells in luteal regression was also explored. Together, these results provided new insight into the spatiotemporal distribution of GC cell types and their transcriptomic features during ovarian development.

## 2 MATERIALS AND METHODS

### 2.1 Animals

Female Wistar rats were purchased from Charles River Laboratories (Beijing, China). Animal handling was performed in accordance with the Guide for the Care and Use of Laboratory Animals published by the US National Institutes of Health. The rats were housed in an environmentally controlled room and had free access to food and water. One 16‐week‐old rat in good condition in the estrus stage was used to perform the experiment after continuous observation of three regular cycles. For the same rat, one retrieved ovary was processed for spatial transcriptomic analysis and the other ovary was handled for scRNA‐seq data analysis. Animal studies were approved by the Zhengzhou University Animal Research Ethics Board (licence no. 2021‐KY‐0054‐002).

### 2.2 Tissue processing

After confirmation of regular cycling, the rat ovaries were retrieved. For the same rat, one retrieved ovary was processed for spatial transcriptomic analysis and the other ovary was handled for scRNA‐seq data analysis.

### 2.3 Ovary sections preparation for spatial transcriptomic analysis

Ovary was rinsed in cold phosphate‐buffered saline (PBS), cryo‐preserved in TissueTek optimal cutting temperature compound (VWR International), and stored at −80°C in an airtight container, as recommended by the manufacturer protocol (10× Genomics, Visium Spatial, CG000240 Rev C). To generate representative sections for the whole ovary, four evenly distributed sections through the ovary were selected for sequencing.

### 2.4 Ovary single‐cell preparation for scRNA‐seq analysis

The rat ovary was chopped using scalpels into pieces of ∼0.3 mm^3^ and digested in Dulbecco's Modified Eagle Media: Nutrient Mixture F‐12 (DMEM/F12, Thermo Fisher Scientific) containing 5% Fetal Bovine Serum (FBS, HyClone, Cytiva), 1 mg/mL collagenase IA (Sigma‐Aldrich), 50 μg/mL Liberase and 1000 U DNase I (Roche, Sigma‐Aldrich) in a shaking 37°C incubator for 40 min 220 rpm. Digestion was stopped with a medium containing 10% FBS and the cell suspension was centrifuged for 7 min on 300 × g. Discard the supernatant and cells were resuspended in Dulbecco's Phosphate Buffered Saline (DPBS, pyg0021, Boster), 2% FBS. The cell precipitate was passed through a 40 μm cell strainer (Millipore, USA). Red cell lysis solution (Solarbio, China) was added to the cell suspension for 5 min. Then the cell suspension was centrifuged for 5 min on 300 × g and resuspended in DPBS, 0.04% Bovine serum albumin (BSA, 1300913761, MACS). The cells were counted and the cell viability was calculated with Trypan Blue (Gibco™, Thermo Fisher).

### 2.5 Single‐cell RNA‐seq library preparation and sequencing

Firstly, dissociated cells were stained with Trypan blue to assess cell number and viability (88.4%). Then, the single‐cell suspension was loaded onto a 10 × Chromium system (10× Genomics). Following GEM generation, cDNA synthesis proceeded using the Single Cell 3′ Reagent Kit v3 (10× Genomics). The amplification of cDNA libraries was amplified by PCR using KAPA Master Mix (kk4600, KAPA Biosystems) with 14 cycles. The cDNA libraries were then fragmented and sequenced on the Illumina NovaSeq 6000 platform using S4 Reagent Kit (Illumina). Demultiplexing of the sample was performed by the ‘mkfastq’ command from Cell Ranger software (10× Genomics, version 1.7.2).

### 2.6 Immunofluorescence

Three female rats' ovarian tissue (8 weeks) were fixed overnight in 4% paraformaldehyde at 4°C. Ovarian tissue was paraffin‐embedded according to standard procedures. The thickness of the paraffin sections was 5 μm. Then sections were deparaffinized using xylene (2 × 15 min), followed by rehydration using gradient alcohol (100%, 95%, 80%, and 70%) and distilled water at 25°C. Antigen retrieval of sections was carried out by high‐temperature treatment at 95°C for 30 min, with sodium citrate buffer (0.01 M, pH 6.0). After cooling, tissue sections were blocked for 1 h at 25°C in a blocking buffer (1% BSA, 0.1% Triton‐X in PBS). Then, sections were incubated overnight at 4°C with primary antibodies. Next day, after rinsing three times with PBS (3 × 15 min), sections were incubated for 1 h with secondary antibodies followed by washing three times with PBS (3 × 10 min). Finally, sections were treated with DAPI (D1306, ThermoFisher) for nuclear visualization and sections were mounted using a fluorescence microscope (Nikon). The primary antibodies including rabbit anti‐StAR (1:100, 8449 T, CST), rabbit anti‐FDX1 (1:200, ab109312, Abcam), and rabbit anti‐ALDH1A1 (1:150, PA5‐95937, ThermoFisher). The secondary antibody was goat anti‐immunoglobulin G (1:500, A‐11008, ThermoFisher).

### 2.7 scRNA‐seq and spatial transcriptomics raw sequence data processing

mRatBN7.2 reference genome data were obtained from the University of California Santa Cruz (UCSC).^20^ Genome Ensembl annotation files were downloaded by the UCSC Table Browser Tool.^21^ CellRanger software (https://support.10xgenomics.com/single-cell-gene-expression/software/downloads/latest) and SpaceRanger software were used to process, align and summarize unique molecular identifier (UMI) counts against rat reference genome for scRNA‐seq pool, and the Visium spatial transcriptomics array, respectively.

### 2.8 scRNA‐seq data analysis

Raw UMI count matrices were imported into R for downstream analysis. To obtain high‐quality data, cells with over 15% UMIs of mitochondrial RNA and below 200 total UMIs were removed. The ‘Scrublet’ software was used to detect the doublets with the parameters expected_doublet_rate = 0.04. All doublets were removed before downstream analysis. For each individual pool, normalization was performed using the ‘SCTransform’ function from Seurat (version 4.0.1)^22^ R package for total UMI counts per cell. The top 2000 highly variable genes were selected based on the mean–variance curve and principal component analysis (PCA) was used to reduce data dimension. Through performing Scree plots, the top 30 PCs (principal components) were calculated for clustering analyses. Cells were then clustered using the Louvain algorithm (resolution = 0.5) for modularity optimization using the K Nearest Neighbors (KNN) graph as input. Finally, the Uniform Manifold Approximation and Projection (UMAP) algorithm was used to visualize these cell clusters.

### 2.9 Spatial transcriptomics data analysis

Raw spot and image data were imported into R for further analysis. Spots not covering the tissue section were filtered out to keep effective spots. SCTransform algorithm^23^ was performed to normalize raw UMI counts for better accounting for the variability in total spot RNA content. Dimensionality reduction was performed using PCA for each slide. Scree plots were used to determine the optimum number of principal components for spot clustering. Clustering was performed using the Louvain clustering algorithm and clusters were visualized using the UMAP algorithm as before. Clusters were visualized in spatial context over Hematoxylin and Eosin staining (H&E) images. Cell population enrichment in each spot was predicted using factor analysis via the ‘FindTransferAnchors’ and ‘TransferData’ functions in Seurat. Spatial feature expression plots were generated with the ‘SpatialFeaturePlot’ function in the Seurat R package.

### 2.10 Identification of differentially expressed genes

We used the ‘Findallmarker’ function from the Seurat R package to determine marker genes for each cell population. Briefly, we defined the genes that expressed for over 10% cell proportion in each cell cluster and log_2_ fold change >0.25 between cells in one cluster and other clusters as marker genes. The markers of sub‐populations were also identified according to the same method. Spatial‐specific expressed genes were identified within the luteum or follicle microenvironment by comparing each interested compartment on tissue sections. DESeq2^24^ was used to screen spatial‐specific expressed genes between interested spatial compartments on tissue sections. Genes with log_2_ fold change >0.5 and adjust *p*‐value < 0.05 were regarded as statistically significant genes.

### 2.11 Cell trajectory analysis

Monocle2 tool was used to construct cell‐type development trajectory among primitive GCs cumulus GCs, mural GCs, and luteum cells.^25^ First, ‘estimateSizeFactors’ and ‘estimateDispersions’ functions were used to normalize the single‐cell gene expression profile. Genes of mean expression >0.1 were selected for downstream analysis. Then, these genes were used for dimensionality reduction. Finally, all cells were ordered based on a pseudo‐time trajectory with DDRTree method.

### 2.12 Gene ontology enrichment analysis

Gene ontology and pathway enrichment analyses were conducted by the ‘clusterProfiler’ R package.^26^ Annotation Dbi R package ‘org.Rn.eg.db’ was used to map gene identifiers. All expressed genes served as background. The results were visualized using ggplot2 R packages.

### 2.13 Ligand–receptor interaction analysis

Ligand–receptor interactions were predicted by CellphoneDB software.^27^ Briefly, the average expression level of ligand and receptor pairs across cell‐type pairs was calculated. Only genes that expressed in more than 10% of cells within each cell type were used for further analysis. We then followed the standard procedure for CellphoneDB to identify the cell communication ligand‐receptor pairs across all cell‐type pairs (https://github.com/ventolab/CellphoneDB). We selected ligand‐receptor pairs with *p* < 0.001 and average expression level >0.2 as significant items.

### 2.14 Transcription factor module analysis

R package ‘SCENIC’^28^ was used to scan active transcription factor modules in GC. Genes detected in fewer than three cells were first filtered. Due to the lack of a rat reference genome in the RcisTarget R package (1.16.0), the mm9 mouse reference genome containing transcription factor motif scores for gene promoters and around transcription start sites were downloaded for further analysis. Previous studies demonstrated that the biological functions and regulatory pathways of homologous genes are highly conserved between species.^29^ Hence, the expression matrices were further filtered to only include homologous genes available in the mm9 mouse reference genome of RcisTarget database. To build a co‐expression module, the expression matrices were used to compute the correlation between two genes by a random forest‐based GENIE3 algorithm. R package ‘SCENIC’ was used to perform transcription factor network analysis to detect co‐expression modules enriched for target genes of each candidate TF from the RcisTarget database. AUCell package (1.16.0) was used to compute the Area Under Curve (AUC) score, which represented transcriptional activity for each TF module in each cell.

## 3 RESULTS

### 3.1 Single‐cell gene expression profiling of rat ovary

**FIGURE 1:**
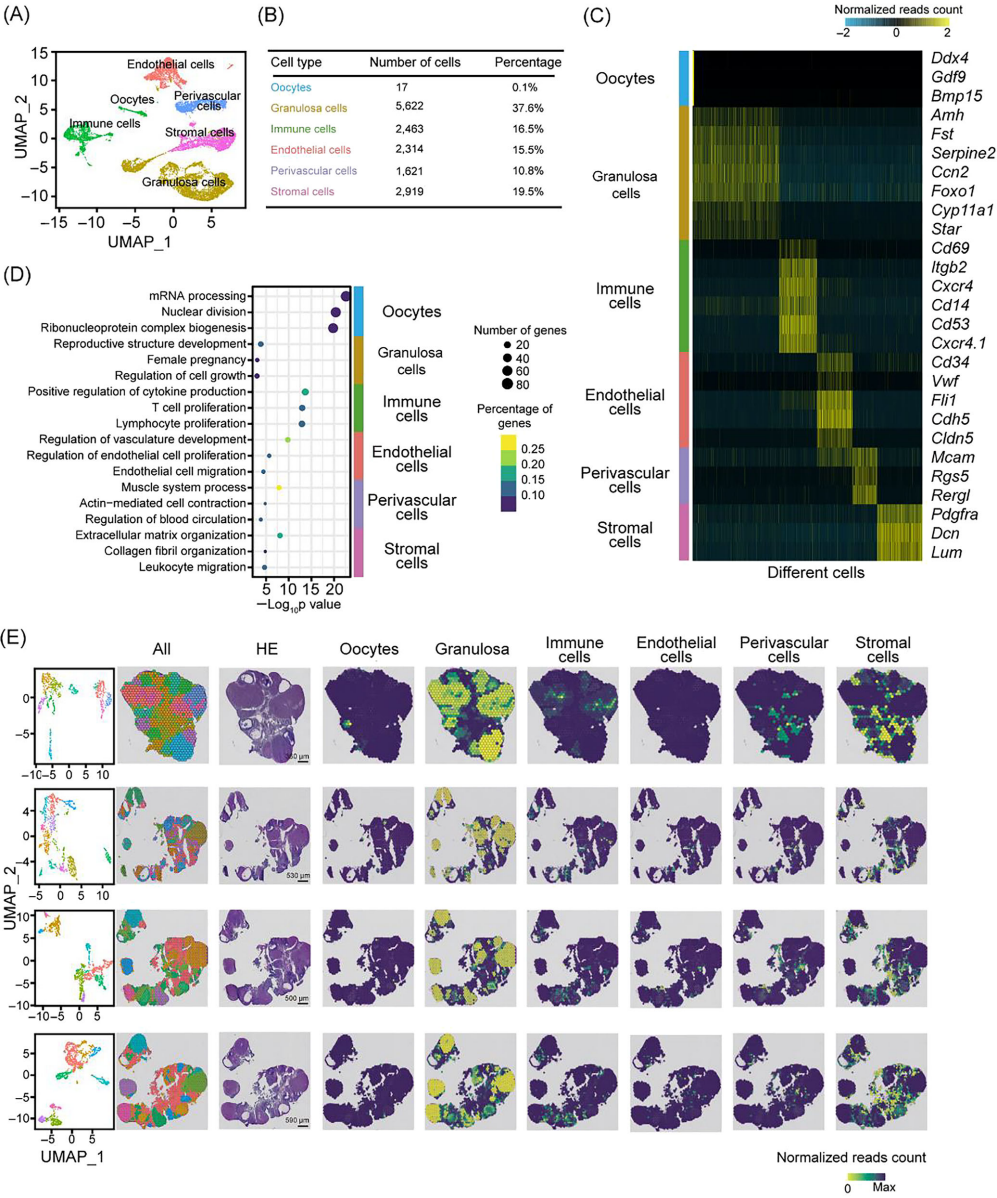
Single‐cell and spatio‐temporal transcriptional atlas of the ovary. (A) UMAP plot of single‐cell transcriptomes of six different cell types from rat ovary. Six main cell types are labelled by distinct colours. (B) Baseline table showing the cell number and percentage of assigned cell types in rat ovary. (C) Heatmap showing the scaled expression levels of cell type‐specific marker genes used for cell annotation. (D) Representative gene ontology terms of stage‐specific gene sets. Circle size and colour represent the number and percentage of genes, respectively. (E) UMAP plot of spot transcriptome clusters from each slide shown on left; clusters are visualized on tissue‐covered slide areas (left). Integration with scRNA‐seq cell type annotations is shown on the right. The last three slides were from sequential sections.

To obtain a single‐cell transcriptome atlas and define the different cell types in the rat ovary, we first performed scRNA‐seq on ovarian tissue based on the 10× Genomics Chromium platform. After quality control, 14,956 cells remained with a median of 1455 expressed genes and coverage of 3946 UMIs per cell (Figure S1A,B). According to the expression features of the known cell‐specific marker genes of distinct cell types, we identified six main ovarian cell types from the dataset, including oocytes (*Ddx4*, *Gdf9*, and *Bmp15*),^18^, ^19^ granulosa (*Fst*, *Amh*, *Serpine2*, *Star*, and *Cyp11a1*),^17^ immune (*Cd69*, *Itgb2*, *Cxcr4*, *Cd14*, and *Cd53*),^17^ endothelial (*Cldn5*, *Vwf*, *Cldn5*, *Cd34*, and *Fli1*),^15^ perivascular (*Mcam*, *Rgs5*, and *Rergl*),^18^ and stromal cells (*Dcn*, *Pdgfra*, and *Lum*; Figures 1A and S1C,D).^18^ As expected, the oocyte‐specific markers showed similar transcriptional signatures between rat and human ovaries.^17^ Intriguingly, in addition to those classic GC markers, we have identified a panel of novel potential markers for GCs, including *Ccn2* and *Foxo1* (Figures 1C and S1D). A cluster corresponding to endothelial cells was identified according to the expression patterns of human endothelial markers such as *CDH5* and *VWF*. But unlike in human ovarian, we found that *Cldn5* also served as a rat endothelial cell marker. Moreover, around 11% of ovarian cells were annotated as perivascular cells, which highly expressed *Mcam*, *Rgs5*, and *Rergl*, with enriched gene signatures associated with both pericytes and smooth muscle cells (Figures 1B,C and S1D).^30^ Additionally, the stromal cells were identified by the expression of diverse mesodermal lineage markers (*Dcn* and *Pdgfra*; Figures 1C and S1D). Consistently, gene ontology analysis results illustrated that those differentially expressed genes in distinct types of cells were highly correlated with their corresponding cell‐type‐specific functions during ovary development (Figure 1D), suggesting that the cell‐type classification based on our data was robust and reliable.

ST analysis was further carried out to explore the spatial distribution characteristics of ovarian cells. We identified 8–12 spot clusters in each slide according to the transcriptional signatures of ST spots, which mapped to the discrete locations (Figure 1E). scRNA‐seq atlas served as a reference to define the single‐cell composition of each spot, thereby spatially localizing all the scRNA‐seq clusters (Figure 1E). As expected, oocytes were enveloped by surrounding GCs, and some of the oocytes at the earlier developmental stages were localized in the vicinity of the ovary surface, confirming that primitive oocytes generally occurred in the ovary cortex. Immune cells predominantly resided in luteum tissue with some distributed in stromal regions (Figure 1E). Moreover, our data from the rat confirmed previous results showing that immune cells including dendritic cells, neutrophils, and macrophages are thought to be involved in luteum remodelling events and cholesterol metabolism.^2^ In contrast, perivascular cells were preferentially exhibited in stromal regions. Since there were few spots judged to be endothelial cells, we did not observe endothelial cells' spatial distribution features obviously. Collectively, we identified six different ovarian cell types, including oocyte and ovarian somatic cells, and depicted both gene expression and spatial distribution features for each cell type.

### 3.2 Differentiation of GCs during follicular development

**FIGURE 2:**
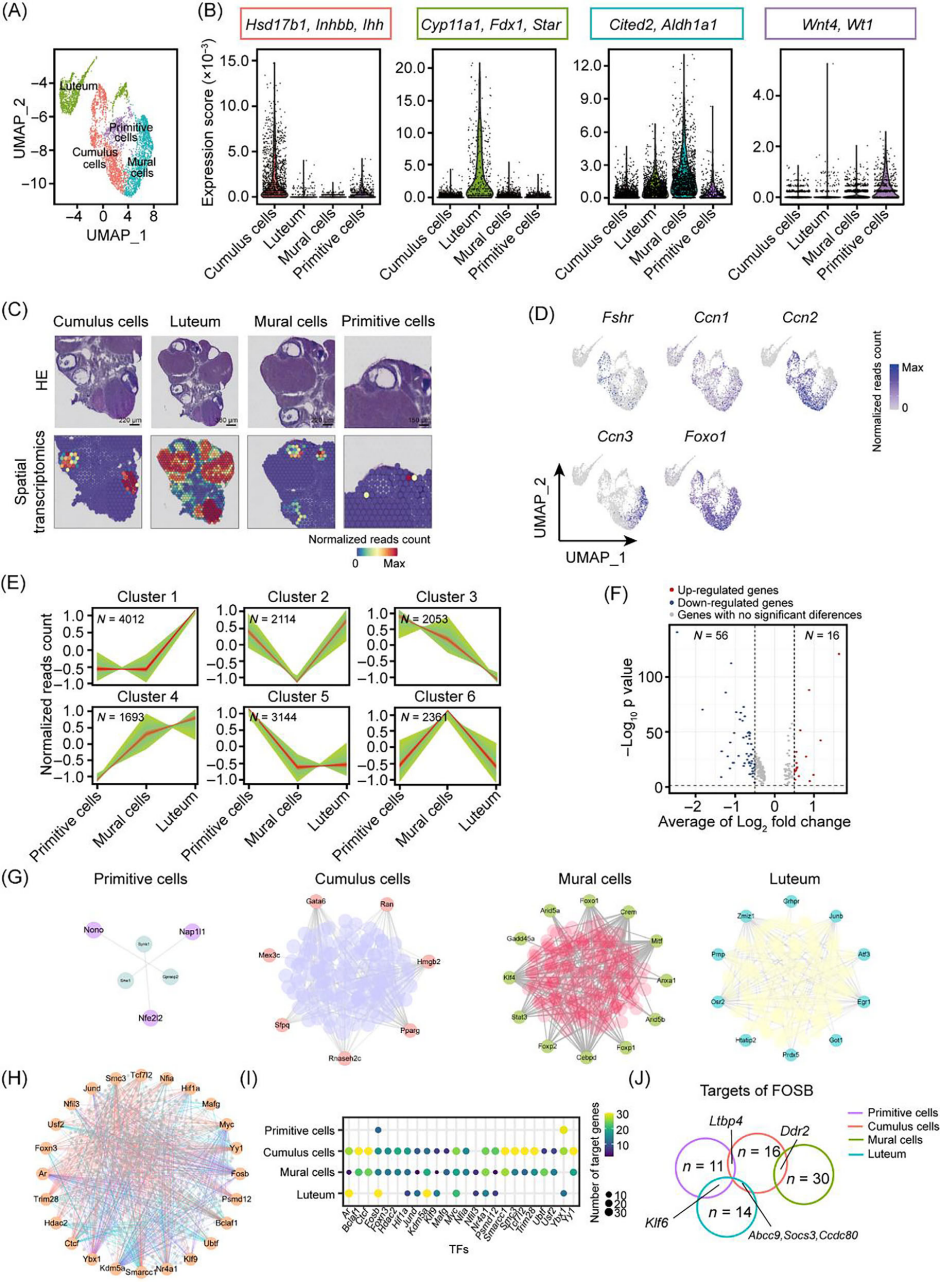
Cataloguing in granulosa cell development. (A) UMAP visualization of four granulosa subclusters labelled by distinct colours. (B) The colour‐coded violin plots showing the average expression scores of signature genes. *Hsd17b1*, *Inhbb*, and *Ihh* for cumulus cells. *Cyp11a1*, *Fdx1*, and *Star* for luteum. Cited2, and *Aldh1a1* for mural cells. *Wnt4*, and *Wt1* for primitive cells. (C) ST spot overlay of cell type predictions for different granulosa subtypes. (D) UMAP plots showing the expression levels of known key genes (*Fshr*, *Ccn1*, *Ccn2*, *Ccn3*, and *Foxo1*) in different granulosa subtypes. (E) Fuzzy c‐means clustering identified six distinct patterns of gene expression. The x‐axis represents three developmental stages, while the y‐axis represents log_2_‐transformed, normalized intensity ratios at each stage. *N* represents the number of genes. Green regions represent the range between minimum and maximum gene expression levels. Red lines represent the mean expression level of the total genes. (F) Volcano plot showing the average RNA abundance change between primitive cells and cumulus cells. (G) Transcription factor (TF) networks of four granulosa subtypes are displayed. The outer and inner nodes of the network represent TFs and their target genes, respectively. (H) Different regulatory networks of shared TFs across four granulosa cell (GC) subtypes. Purple, red, green, and blue lines represent the interactions of primitive cells, cumulus cells, mural cells, and lutetium, respectively. (I) Dot plots showing the number of target genes of shared TFs across four GC subtypes. (J) Venny chart showing the different targets of TF Fosb among four GC subpopulations.

Considering that the oocyte–GC interactions and their microenvironment conditions considerably influence follicle and oocyte growth and maturation,^31^ we then defined the dynamic molecular changes of GCs that take place during follicle development. According to the clustering results, the granulosa population was divided into four compartments including cumulus, luteum, mural, and primitive GCs (Figure 2A,B). The four granulosa populations were further mapped to the tissue sections by Seurat (Figure 2C). Cumulus cells were determined based on the expression levels of marker genes,^17^ such as *Inhbb*, *Ihh*, and *Hsd17b1* (Figures 2B and S2A). Mural cells were identified by *Cited2* which also presented a high expression level in human mural GCs^17^ (Figures 2B and S2A). In addition to some reported mural markers, we found that *Aldh1a1* exhibited high RNA abundance in mural cells which could serve as novel rat mural markers (Figures 2B and S2A). Indeed, both ST data and immunofluorescence results verified that *Aldh1a1* was located in the wall of follicles (Figure S2BC). Earlier studies have demonstrated that FSH is involved in the regulation of follicle development,^32^, ^33^, ^34^ whereas the detailed mechanism remained to be fully revealed. Our results showed that *Fshr* was preferentially expressed at higher levels in cumulus and mural cells, suggesting that FSH may stimulate follicle growth primarily by communicating with these two cell types (Figures 2D and S2F). CCN growth factors, as the downstream genes of hippo signalling that disrupt follicle growth,^5^, ^35^ displayed distinct expression patterns between mural and cumulus cells (Figures 2D and S2F). In contrast to the high *Ccn2* expression in cumulus cells, mural cells had a low *Ccn2*, but a high *Ccn3* expression. Both cumulus and mural presented weak *Ccn1* signals (Figure 2D). These findings suggested that hippo signalling might function through distinct target gene axis among different granulosa subtypes.

The cell cluster with highly expressed stem lineage‐related genes of *Wt1* and *Wnt4* and derived from mouse ovary thecal progenitors^36^ was defined as primitive GCs (Figures 2B and S2A). Further trajectory analysis also showed that primitive cells were the common ancestor of cumulus and mural cells, confirming the accuracy of cell identification (Figure S2G). Importantly, we observed a cell cluster with low expression of conserved known GC marker *Foxo1* ^37^ which was located in the luteum region (Figure 2C,D). Moreover, this cluster was deposited behind the mural sub‐population according to the trajectory analysis, suggesting that it may evolve from mural cells (Figure S2G). So, we defined these cells as luteal populations based on the previous findings that the luteal compartment was differentiated from mural cells after ovulation.^2^ Genes specifically expressed in the corpus luteum were then selected as marker genes, such as *Cyp11a1*, *Fdx1*, and *Star* (Figures 2B and S2B). Previous studies also showed that these three genes are related to lipid metabolism, which is in line with luteum function.^38^, ^39^ Notably, immunofluorescence further validated that *Fdx1* and *Star* were specifically expressed in the luteal region, corresponding to their spatial distribution pattern (Figure S2B,D,E). Next, we identified subgroup‐specific genes for all GC compartments and found that those subgroup‐specific genes were highly correlated with corresponding cell function (log_2_ fold change >0.25, *p* < 0.05; Figure S2H). Additionally, the gene expression patterns were also determined during granulosa development (Figure 2E). These genes were clustered into six patterns that meet their respective functional characteristics in the primitive‐mural‐luteal axis, suggesting that granulosa may be subjected to different regulatory patterns during development (Figures 2E and S2I). Moreover, we also compared the gene expression changes in the primitive‐cumulus axis. Difference gene expression (DEG) analysis between primitive and cumulus cells showed that cell growth‐related genes were up‐regulated and stem cell‐related genes were down‐regulated in cumulus, suggesting that cumulus cells proliferate massively to support the oocyte development (Figures 2F and S2J). Altogether, we identified four types of GCs and resolved their differentiation directions during rat follicular development.

To further characterize the transcriptome features of different GCs, a cell type‐specific transcriptional regulatory network (TF net) was constructed by SCENIC. As shown in Figure 2G, the TF net of primitive cells had the fewest interaction nodes of TFs and targets (*N* = 3) compared with other GC types (*N* = 7–13). Thus, we inferred that primitive cells were relatively quiescent or transcriptionally inactive. In order to understand the differences in the regulation of shared transcription factors among distinct GC subsets, we constructed a network of differential regulatory models based on shared TF in GCs (Figure 2H). Importantly, we found that *Fosb* was a shared TF across all GC subsets (Figure 2I). However, its downstream target genes varied in distinct GC sub‐populations, denoting that *Fosb* was a key TF during all follicle development stages and that the regulatory modes of *Fosb* shifted during GCs differentiation (Figure 2I,J). In addition, primitive GC cells shared the fewest number of TFs with other GCs, further indicating that it was in a transcriptional quiescent state (Figure 2I; *N* = 2). Altogether, these results delineate the GC developmental trajectories and characterize the molecular changes in GC subtypes during follicle development.

### 3.3 Cellular crosstalk between granulosa and other ovary cells

**FIGURE 3:**
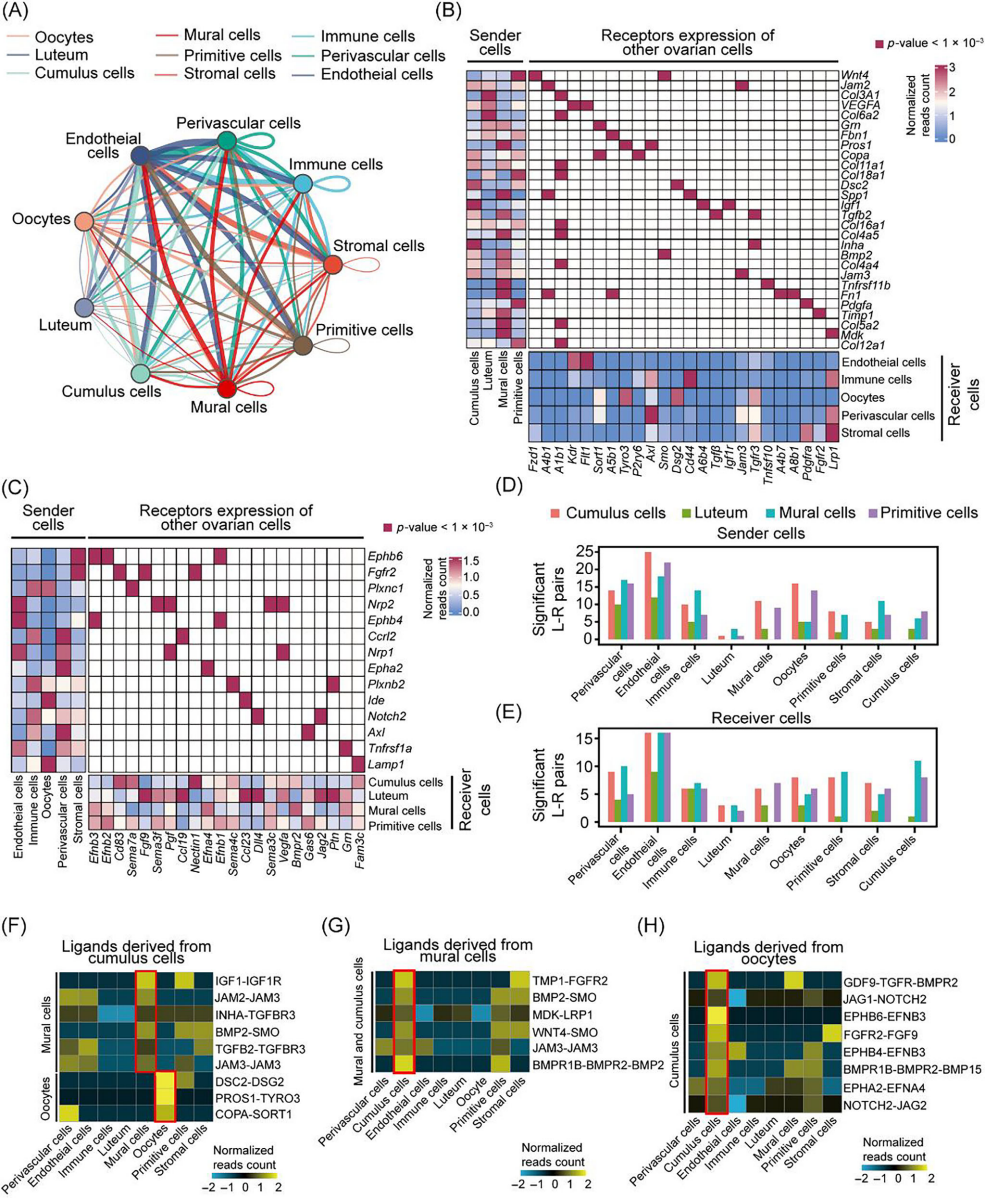
Interaction between granulosa cells and other somatic ovarian cells during follicular development. (A) The cellular communication network of four granulosa subtypes with ovarian cells. (B) Heatmap showing the average expression of cellphoneDB predicted ligands expressed by granulosa cells (GCs; left), ligand‐matched receptors expressed by ovarian cells (bottom), and the significantly enriched ligand‐receptor pairs between granulosa sub‐populations and ovarian cell type pair (middle) from scRNA‐seq data. (C) Heatmap showing the expression of cellphoneDB predicted ligands expressed by ovarian cells (left), ligand‐matched receptors expressed by GCs (bottom), and the significantly enriched ligand‐receptor pairs between ovarian cell types and granulosa sub‐population pair (middle) from scRNA‐seq data. (D,E) Bar plots of significantly enriched ligand–receptor (L–R) pairs (*p* < 0.001). Ligands were expressed in GCs subtypes and receptors were expressed across immune cells, perivascular cells, and endothelial cells (D). Receptors were expressed in GCs subtypes and ligands were expressed across immune cells, perivascular cells, and endothelial cells (E). (F–H) Heatmap showing the average expression of L–R pairs for which ligands derive from cumulus and receptors are expressed in oocyte and mural GC (F), ligands derive from mural and receptors are expressed in cumulus GC (G), and ligands derive from the oocyte and receptors are expressed in cumulus GC (H).

To investigate the interaction between granulosa and environmental cells, scRNA‐seq, and ST datasets were integrated to characterize the crosstalk signals between GCs and other ovary cells. Based on the database of ligand–receptor pairs from CellphoneDB software, all significantly enriched L–R pairs were identified and the ligand–receptor interaction network was established (Figure 3A−C). Interestingly, we found that endothelial cells participate in extensive paracrine interactions with all GC sub‐populations with a larger number of L–R pairs, suggesting that they may be responsible for signal conditioning during follicle development (Figure 3D,E). Within a follicle, the cumulus is a ‘relay station’ for mural and oocyte signalling communication.^5^ Thus, we focused on the crosstalk between cumulus and mural or oocyte cells. As shown in Figure 3F,G, we further identified several ligand–receptor signalling pathways with spatial proximity among GC sub‐populations, such as the ligand–receptor pairs of IGF1‐IGF1R and INHA‐TGFBR3 in cumulus to mural cells, TIMP1‐FGFR2 and MDK‐LRP1 in the mural to cumulus cells (Figure 3F,G and S3A,B). Importantly, several novel signals of oocytes to cumulus were identified, including JAG1‐NOTCH2 and FGF9‐FGFR2 (Figures 3H and S3A). Overall, we drew the potential crosstalk between granulosa and other ovary cells, and identified novel intercellular signals that may be involved in the regulation of follicle development.

### 3.4 Unique gene‐expression pattern of three subtypes of cumulus at sequential and stepwise developmental stages

**FIGURE 4:**
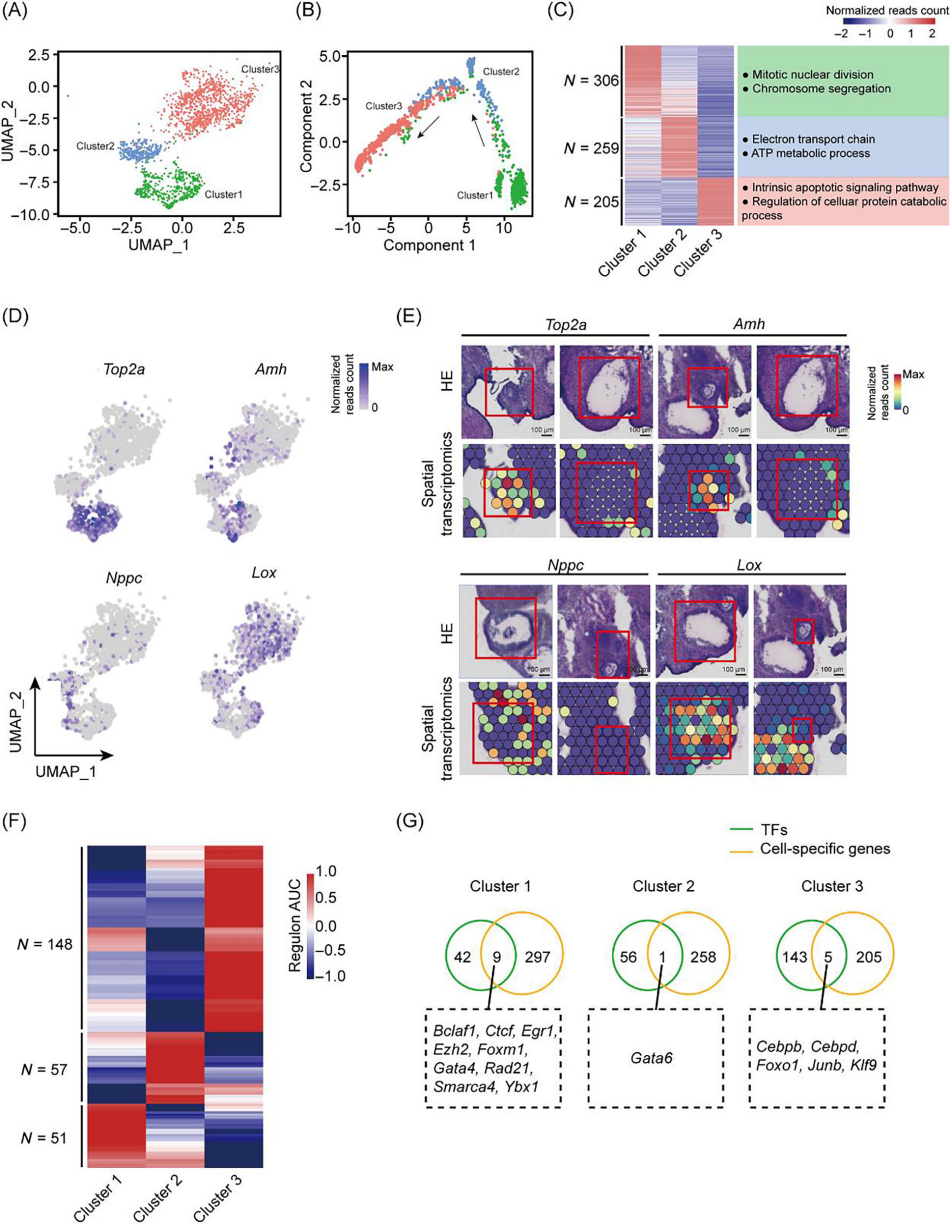
Cumulus state transition during follicle development. (A) UMAP visualization of cumulus subclusters labelled by distinct colours. (B) Trajectory analyses of cumulus cells using the monocle algorithm are shown over UMAP embedding, coloured by cell clusters. Arrow represents developmental direction. (C) Heatmap showing the scaled expression levels of differential expressed genes among different cumulus subclusters. Representational GO terms also are shown on the right. (D and E) UMAP plot and spatial feature plots showing the expression levels of known marker genes (*Top2a*, *Amh*, *Nppc*, *Lox*) among distinct cumulus compartments. The H&E images derive from Sections 1 to 4. (F) Clustered heatmap of TF regulons by SCENIC analysis (*n* = 256 regulons) across cumulus sub‐populations. (G) Venn diagram illustrating the overlap between subtype‐specific TFs and differentially expressed genes among different cumulus subtypes.

To explore the gene expression dynamics of cumulus during folliculogenesis, we performed unsupervised analysis and subsequently identified three subtypes of cumulus cells (Figure 4A). Trajectory analysis results revealed that cluster 1 was distributed at the root of the pseudo‐time trajectory (Figure 4A,B). Then, functional enrichment analysis for specifically expressed genes was performed in each cumulus subtype. We found that genes in Cluster 1 at the end of the pseudo‐time trajectory were significantly enriched in cell proliferation‐related items (Figure 4B,C). So, we inferred that cluster 1 mainly consists of GCs from early antral follicles. As essential genes for follicular development, *Top2a*, *Amh*, *Nppc*, and *Lox* were differentially expressed in distinct cumulus subtypes (Figure 4D,E). Subtype cluster 1 showed specific expression of proliferation‐related gene *Top2a* and high expression of AMH which was reported to locate in early antral follicles or small antral follicles,^40^ confirming that cell cluster 1 is associated with the rapid growth of follicles prior to ovulation (Figure 4D,E). *Nppc* arresting oocytes meiotic^41^ was preferentially expressed in subtype cluster 2, pointing out that those cells were in the follicle before ovulation (Figure 4D,E). *Lox*, which acts as ovulation‐related genes,^42^ was specifically up‐regulated in the cumulus subtype from Cluster 3, indicating that the cells in this cluster were at the stage of ovulation (Figure 4D,E). Thus, we have traced the sequential and stepwise developmental trajectory of folliculogenesis for cumulus cells from Clusters 1 to 3.

Then, we identified active transcription factors in the three sequential cumulus stages. The unsupervised analysis result showed that all transcription factors could be divided into three compartments corresponding to cumulus classification (Figure 4F). To identify the potential master regulators involved in the stage‐to‐stage transition in cumulus, we intersected these transcription factors with the corresponding cumulus subtype‐specific genes. Fifteen transcription factors were identified to be the potential critical regulons essential for cumulus transition, such as *Bclaf1* in Cluster 1, *Gata6* in Cluster 2, and *Cebpb* in Cluster 3 (Figures 4G and S4). *Bclaf1* is required for smooth muscle lineage differentiation during lung development.^43^ In the pancreas, GATA6 binds the promoter of the digestive enzyme genes *Rbpjl* and *Mist1* facilitating acinar differentiation.^44^ Similarly, *Cebp3* is also involved in cell development and differentiation, and a previous study has shown that it can regulate osteoblasts differentiation through the WNT/ β‐Catenin pathway.^45^ Follicular development is a step‐by‐step orchestrated process containing the programmed expression of gonadal hormones and other environmental factors,^5^, ^31^ which probably affected the activity of these TFs and thus regulates cumulus transition. In total, our findings revealed the stage‐specific transcriptional programs underlying cumulus transition in rat ovaries.

### 3.5 Immune cells involving luteal regression

**FIGURE 5:**
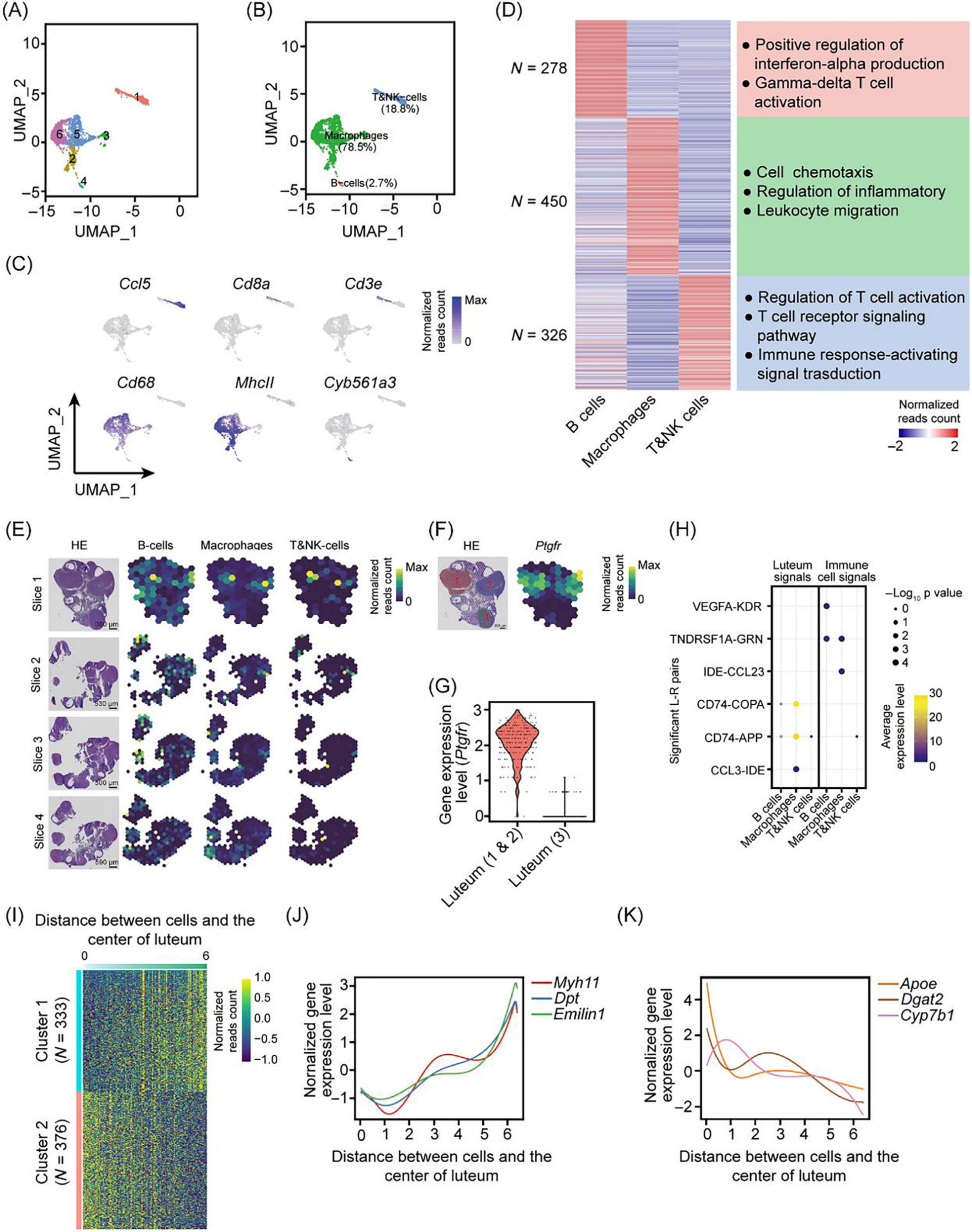
Immune cells are involved in the process of luteal regression. (A and B) UMAP cluster map revealing six specific immune cell subtypes (A) and three mainly immune cell subtypes (B). (C) Heatmap showing the scaled expression levels of differential expressed genes among immune subtypes. Representational gene ontology terms also are shown on the right. (D) UMAP plots showing the expression signatures of different immune subtypes. (E) ST spot overlay of cell type predictions for immune compartments in two consecutive sections. (F) Spatial feature plots showing the expression levels of *Ptgfr* in section 1. (G) Violin plot showing the expression level of *Ptgfr* between luteum 1 or 2 and 3. (H) Dot plot showing the average expression levels and significance of the ligand–receptor (L–R) pairs in B cells, Macrophages, and T&NK cells. (I) Heatmap showing the two genes' expression pattern with gradient increased (Cluster 1) or declined (Cluster 2) expression levels from the centre to outer in luteum regions. (J,K) The distribution of gene expression of representative spatial‐restricted genes in Cluster 1 (J) and Cluster 2 (K).

To interrogate the composition of immune cells in the ovary, we first separated the immune cells into 6 compartments based on clustering analysis (Figure 5A). Macrophage signatures were prevalently expressed in Populations 2, 3, 5, and 6, and these cells accounting for the highest proportion of immune cells were defined as macrophages (Figure 5B,C). Cells in Population 1 presented a high expression level of both T cell markers (*Cd8a* and *Cd3e*)^46^ and NK cell marker *Ccl5*,^47^ which were categorized into a T&NK‐cell population (Figure 5B,C). Population 4 with minority cells was defined as B cells according to the expression pattern of *Cyb561a3* ^48^ (Figure 5B,D). As expected, the cell type‐specific genes were enriched in their corresponding functional terms (Figure 5C). Previous studies demonstrated that immune cells may participate in luteum remodelling.^2^ To further study its critical role in follicular development, immune cells were mapped to HE slides by integration of scRNA‐seq and ST data. Interestingly, immune cells were only selectively distributed in some luteal regions (Figure 5E).

We then analysed the DEGs between luteum regions with high or low immune cell enrichment. We found that the up‐regulated genes were highly related to lipid metabolism in the luteum, which lacks immune cells, but associated with immune response in immune cell‐enriched luteum (Figure S5A,B). A high expression level of *Ptgfr* was predominantly detected in the luteum with high enrichment of immune cells (Figure 5E−G), denoting the initiation of the luteolysis process.^49^ Therefore, the distinct distribution pattern of immune cells in luteum regions suggests a potential function of immune cells in the luteolysis process. Next, intercellular communication signals were calculated between immune and luteal cells (Figures 5H and S5C). Macrophages exhibited maximum amounts of L–R pairs with luteal cells and the highest abundance in luteum regions, thus representing the key cell type likely involving luteolysis (Figure S5C). In addition, we found obvious spatial heterogeneity of gene expression in the luteum (Figure 5I). The expression of lipid metabolism‐associated genes, including *Apoe*, *Dgat2*, and *Cyp7b1*, gradually declined from the centre to the outer of the luteum, while ExtraCellular Matrix (ECM) formation‐associated genes of *Myh11*, *Dpt*, and *Emilin1* showed an increased expression tendency, indicating that the spatially restricted gene expression pattern in luteum was corresponding to its function (Figures 5J,K, and S5D, E). Overall, these results highlight the important roles of immune cells in luteal regression with spatial heterogeneous expression features in the luteum.

## 4 DISCUSSION

Although various cell types have been identified in the mammalian ovary by scRNA‐seq data,^15^, ^17^, ^18^, ^19^ spatiotemporal regulatory mechanisms during folliculogenesis remain unclear. The integration of single‐cell and spatial information in this study enables us to interrogate the heterogeneity of spatiotemporal transcriptional programs in distinct ovarian compartments during ovary development. We identified six types of gene‐expression signatures in the rat ovary and further defined the differentiation hierarchies of developing GC subtypes with known and several previously unreported luteal markers. Moreover, we described three sequential cumulus states during follicle development and determined the key transcriptional programs. Finally, we highlighted the immune cells, especially macrophages that were related to luteal regression and heterogeneous luteal gene expression.

Regarding to the cellular composition of the rat ovary, we identified six ovarian cell types containing oocytes and five somatic compartments based on their unique scRNA‐seq molecular signatures and spatial information. The canonical markers of distinct ovary cell types derived from humans or mice also showed specific expression in the corresponding cell subsets of the rat, illustrating the conserved molecular characteristics among different species and the reliability of our analysis data. Although the majority of known cell‐specific marker genes showed conserved expression in both rats and humans, species‐specific gene expression should be taken into account in cell clustering. Especially, the variable genes discovered in GCs from our dataset could be potentially used as candidate cell‐type‐specific markers. Additionally, in our data, 17 oocytes were identified based on the expression of *Ddx4*, *Gdf9*, and *Bmp15* ^50^, ^51^, ^52^ (Figures 1C and S1C), which was similar to previously reported results that 18 oocytes were obtained,^19^ hinting that more large scale and precise sequencing techniques should be established to obtain enough oocytes and surrounding cells for in‐depth exploration of ovarian development. Due to luteal cells being transformed from mural GCs during follicular development,^53^ it is difficult to distinguish these two cell types only by gene signatures. Through combining single‐cell and ST data, we successfully identified luteal cells based on several lipid metabolism‐related markers in our data such as *Fdx1*, *Fdxr*, and *Cybpa11*, which correspond to the physiological functions of luteum (Figure 2A–D). These luteal signatures will provide a reference for future luteal cell identification.

Most previous studies described the diversities of GCs during follicle development in humans and mice,^54^, ^55^, ^56^ but the regulatory network and molecular mechanisms underlying GC development in ovaries remain largely unknown. By using scRNA‐seq, we delineated comprehensive transcriptional program changes of GCs in rat ovaries (Figure 2). It is worth noting that the crosstalk between oocytes and GCs, an important compartment of the follicle, plays an important role in follicle growth and development.^2^, ^5^, ^11^ For example, oocytes were aroused by GCs through KIT‐KITL signalling. Then, BMP15 and GDF9 produced by oocytes spread into GCs, leading to the activation of growth signalling SMAD in GCs.^57^ In our data, we identified some novel signal pathways such as JAG1‐NOTCH2 and FGF9‐FGFR2 between oocytes and GCs.^58^, ^59^, ^60^, ^61^ The Notch pathway is a highly conserved juxtracrine signalling participating in many cellular processes, including differentiation and proliferation.^62^ Our data showed that *Notch* mRNA was specifically expressed in the cumulus, suggesting that oocytes may stimulate cumulus development through JAG1‐NOTCH2 signalling (Figure 3H). Both cumulus and mural cells expressed *Fgfr2*, which is highly associated with proliferation.^63^ Therefore, we inferred that the *Fgf9* ligand expressed in oocytes might interact with the *Fgfr2* receptor to activate growth signalling in GCs, supporting the findings that growth signals of GCs are primarily initiated by oocytes.^31^

Previous studies have expounded on how GCs converge to regulate follicle development.^18^ However, much less is known about the dynamic of gene expression and state transition in granulosa during follicle development. In this study, we first distinguished three consecutive stages of cumulus cells, associating with their corresponding functional characteristics, evidenced by the certain TFs were specifically expressed in distinct stages of cumulus cells (Figure 4A–C). Moreover, mural cells differentiate into luteal cells after ovulation, which indirectly proves that the transition of GC states occurs during follicle development.^53^

Recent work demonstrated that immune cells are abundant in the developing corpus luteum^64^, ^65^ and are thought to facilitate tissue remodelling events as well as control steroidogenic function.^66^, ^67^ However, other studies argued that immune cells directly participate in the loss of steroidogenesis and the demise of luteal cells and tissue.^68^, ^69^ We found that immune cells are mainly located in the inside luteum, supporting an association between immune and luteal cells. Meanwhile, we observed that immune cells contain B, T&NK, and macrophage cells in rat ovarian, and they are closely related to corpus luteum regression (Figure 5), which is supported by the finding that immune cells mainly reside in the resolving corpus luteum and less in the developing corpus luteum. Further comprehensive studies are needed to explore whether immune cells are involved in luteum remodelling. Besides, we observed an inconsistent abundance of B/macrophage cells between single‐cell and ST sequencing data (Figure 5B,E). This may cause by the distinct origins of cells that are used for these two types of sequencing techniques, and the bias of integrated analysis.^70^ Hence, developing new techniques to capture single‐cell and ST simultaneously will further enhance the precision of omics analysis. Moreover, our study showed that macrophages communicate with luteal cells through several signal pathways such as Tnfrsf1a/Grn, Ide/Ccl23/3, Cd74/Copa, and Cd74/App (Figure 5H). We inferred that macrophages participate in luteum resolving through these signal pathways. Due to the low number of other immune cells being detected, few intercellular signalling was identified between luteal and other immune cells. Therefore, the crosstalk analysis among other ovarian immune cell types remains to be resolved.

Activation of the LH receptor in follicular cells by the preovulatory LH surge causes ovulation and rapid initiation of a terminal differentiation program, which makes the ovulated follicle into a corpus luteum through a process termed luteinization.^71^ The corpus luteum plays important roles in regulating the estrous cycle and maintaining pregnancy.^72^ This function is carried out largely by progesterone, which is the main steroid synthesized by this transient endocrine gland.^53^ However, the molecular foundation of luteum development remains largely unknown. Our data revealed transcriptome heterogeneity of luteal at the single‐cell level through scRNA‐seq and ST (Figure 5). What's more, we found that genes related to hormone metabolism are mainly occurred inside the corpus luteum, whereas genes related to ECM are preferentially expressed outside the corpus luteum (Figures 5I and S5D,E). These findings provide a new perspective for our understanding of luteal development.

In summary, by combining the scRNA‐seq and spatial transcriptomic sequencing, we established the first comprehensive spatiotemporal transcriptomic atlas of rat ovarian, which broadens our understanding of cell identities and follicle formation in the mammal ovary. Importantly, it provides in‐depth knowledge about the molecular mechanisms underlying ovary development in rats, which would be valuable not only as the reference resources, but also for a better understanding of the molecular basis of ovary disease.

## AUTHOR CONTRIBUTIONS

*Conceptualization and design*: Yingpu Sun, Yun‐Gui Yang, Xiao Han, and Lanlan Fang. *Experiment performance*: Yanjie Guo, Lanlan Fang, and Jung‐Chien Cheng. *Bioinformation analysis*: Yong Shi. *Experiment assistance*: Guanshen Cui. *Bioinformation assistance*: Xiao Han, Jiayi Zhou, and Ying Wu. *Visualization*: Yong Shi and Xiao Han. *Article writing*: Yong Shi, Yanjie Guo, Xiao Han, and Lanlan Fang. *Article editing*: Yong‐Liang Zhao. *Supervision*: Yingpu Sun and Yun‐Gui Yang.

## FUNDING INFORMATION

This study was supported by the National Key R&D Program (2019YFA0110900), the Strategic Priority Research Program of the Chinese Academy of Sciences (XDA16010501), National Natural Science Foundation of China for the International (Regional) Cooperation and Exchange Projects (81820108016), and the Operating grant from the National Natural Science Foundation of China (32070848).

## CONFLICT OF INTEREST STATEMENT

The authors declare that they have no conflict of interest.

## Supporting information


**Data S1.** Supporting information.

## Data Availability

High‐throughput sequencing data and corresponding HE image data that support the findings of this study have been deposited at Genome Sequence Archive and OMIX (https://ngdc.cncb.ac.cn/omix), China National Center for Bioinformation/Beijing Institute of Genomics, Chinese Academy of Sciences^73^ under the accession number CRA008987 and OMIX002408‐01 linked to the project PRJCA013022.
