# Supplementary material for: A spatiotemporal gene expression and cell atlases of the developing rat ovary
Source: Cell Prolif. 2023 Jun 13;56(12):e13516. doi: 10.1111/cpr.13516 (PMC10693188; doi:10.1111/cpr.13516)
Supplement: Supplementary file 1 — Data S1. Supporting information. [file CPR-56-e13516-s001.docx]

**Supplementary Data**

**A spatiotemporal gene expression and cell atlases of the developing rat ovary**

Yong Shi | Yanjie Guo | Jiayi Zhou | Guanshen Cui | Jung-Chien Cheng |Ying Wu | Yong-Liang Zhao | Lanlan Fang^#^ | Xiao Han^#^ | Yun-Gui Yang^#^ | Yingpu Sun^#^

**Supplementary Figures**


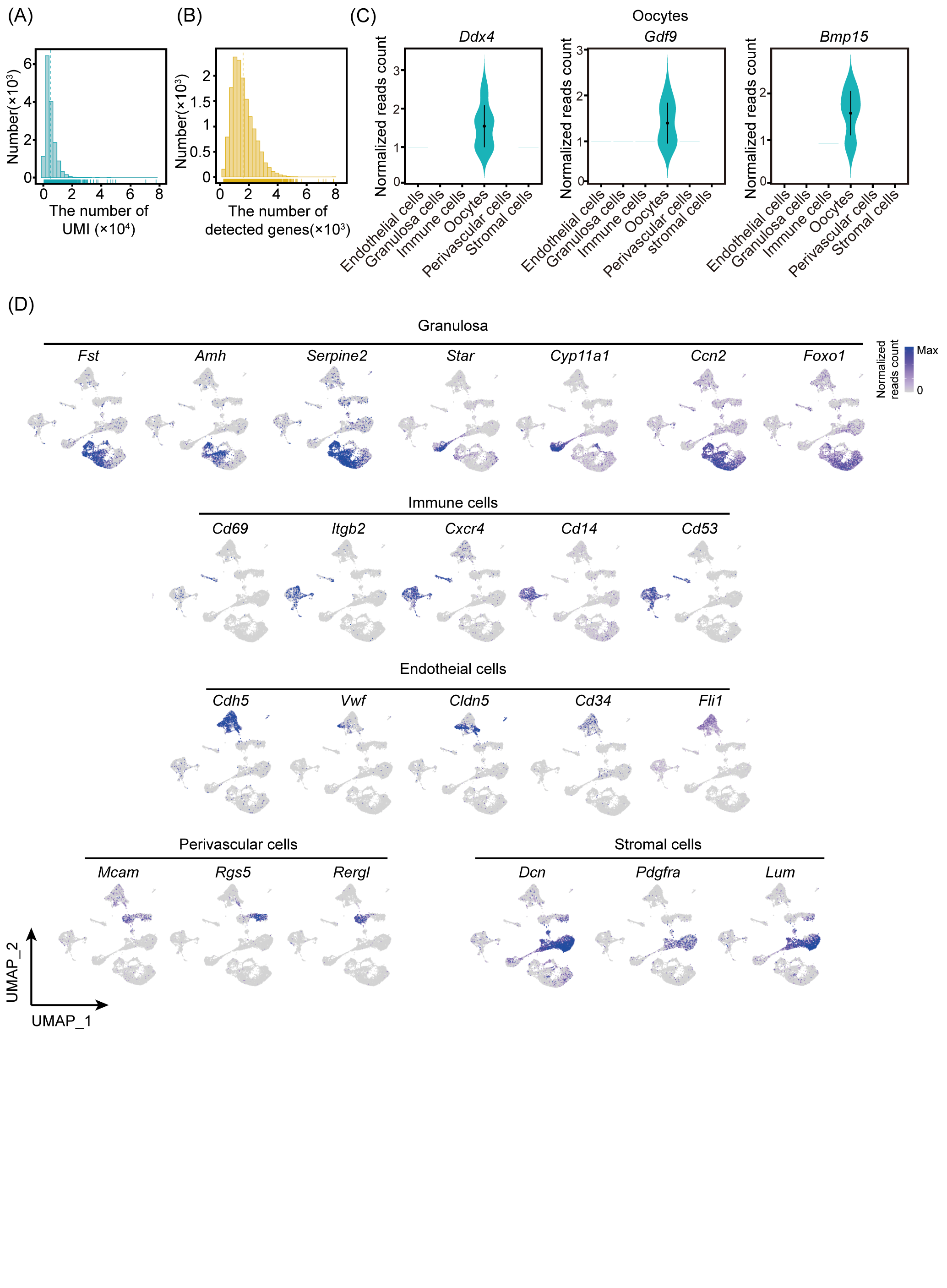


**FIGURE S1 Overview of quality control of single-cell RNA-Seq.** (A) Histogram of total UMI counts per cell. (B) Histogram of the number of detected genes per cell. (C) Violin plots showing the expression of oocyte marker genes *Ddx4*, *Bmp15*, and *Gdf9* among six main ovarian cell types. (D) UMAP plots showing the expression levels of known and newly identified marker genes in distinct ovarian cells.


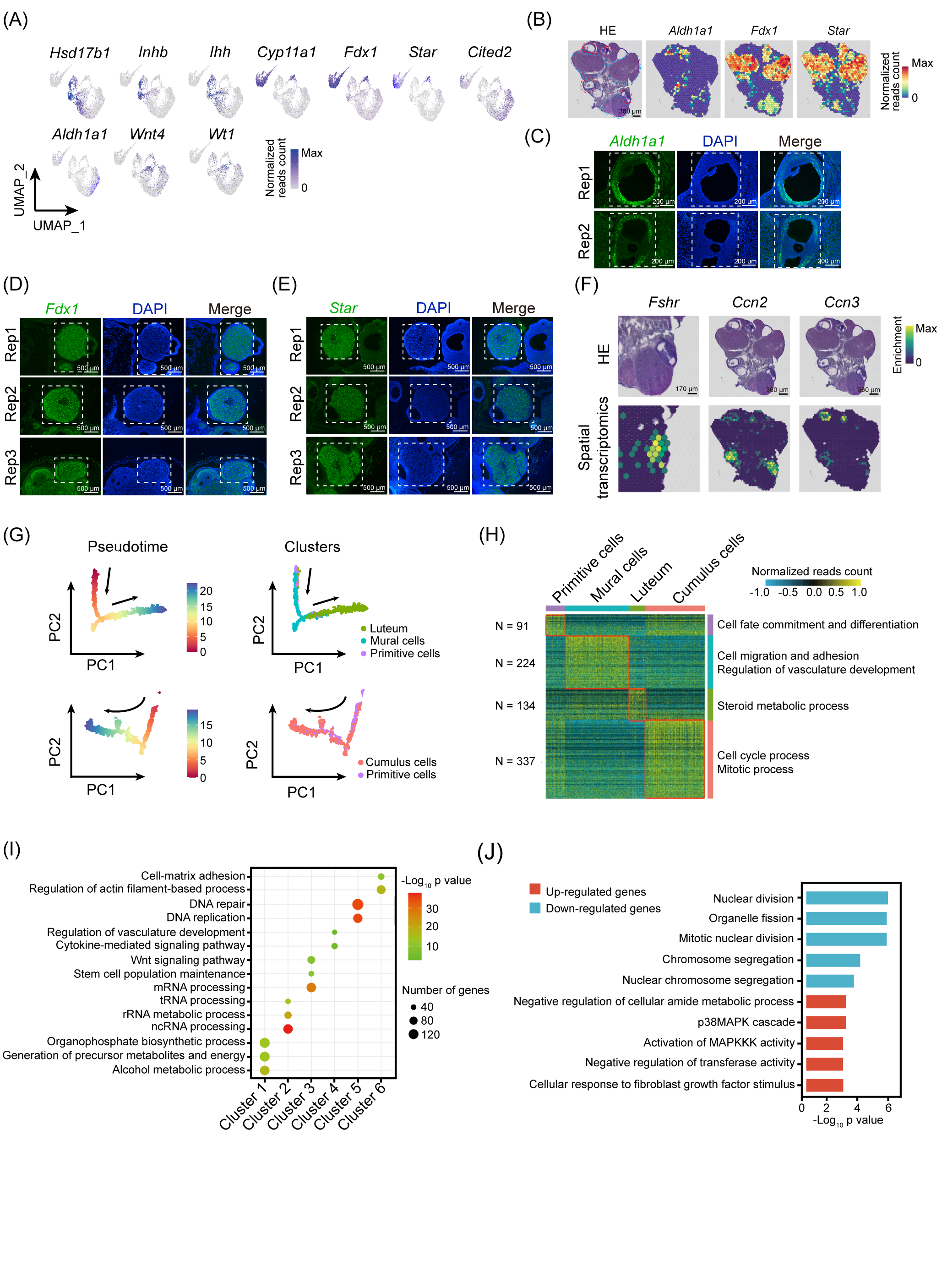


**FIGURE S2 Development of granulosa compartments.** (A) UMAP plots showing the expression levels of signature genes for distinct granulosa compartments. (B) ST overlay of marker genes *Aldh1a1*, *Fdx1*, and *Star* in section 1. Red and blue dash lines represent follicles and luteum, respectively. (C−E) Immunofluorescence staining of mural marker gene *Aldh1a1* (C) and luteal marker genes *Fdx1* (D) and *Star* (E). (F) Spatial feature plots showing the expression pattern of known key genes in different granulosa subtypes. (G) PCA plots showing granulosa cell trajectory labeled by cell subtypes and pseudo-time. (H) Heatmap showing the scaled expression levels of signature genes which are differentially expressed among granulosa subtypes (left). Representative GO terms also are shown (right). (I) Representative GO terms of six distinct gene patterns for primitive-mural-luteal GC axis. (J) Representative GO terms of differentially expressed genes between primitive and cumulus cells.


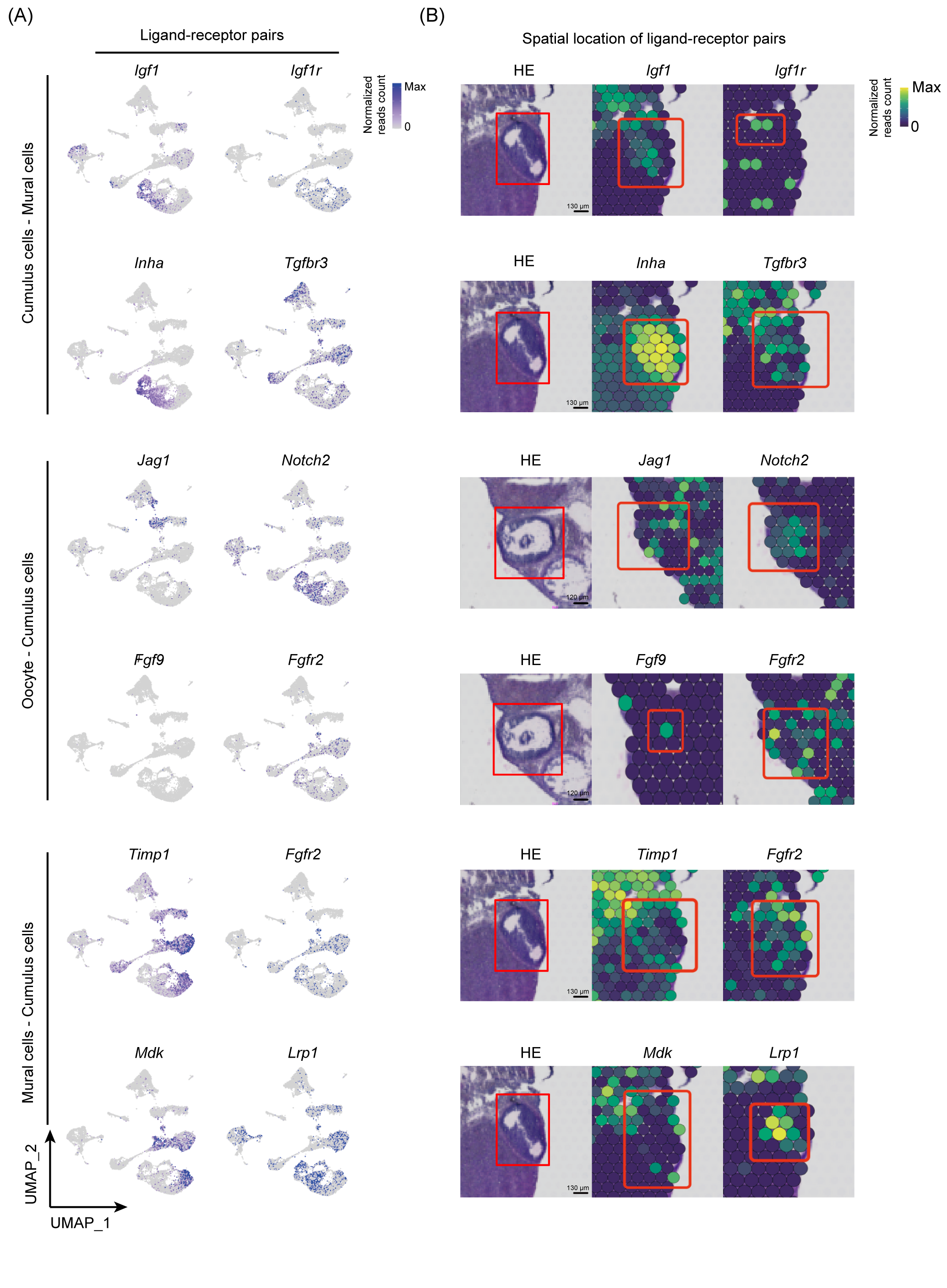


**FIGURE S3 Ligand-receptor expression in granulosa cells and oocytes.** (A) UMAP plots showing the gene expression levels of ligand-receptor pairs. (B) Spatial feature plots showing the ligand-receptor co-localization. All H&E images were from section 1.


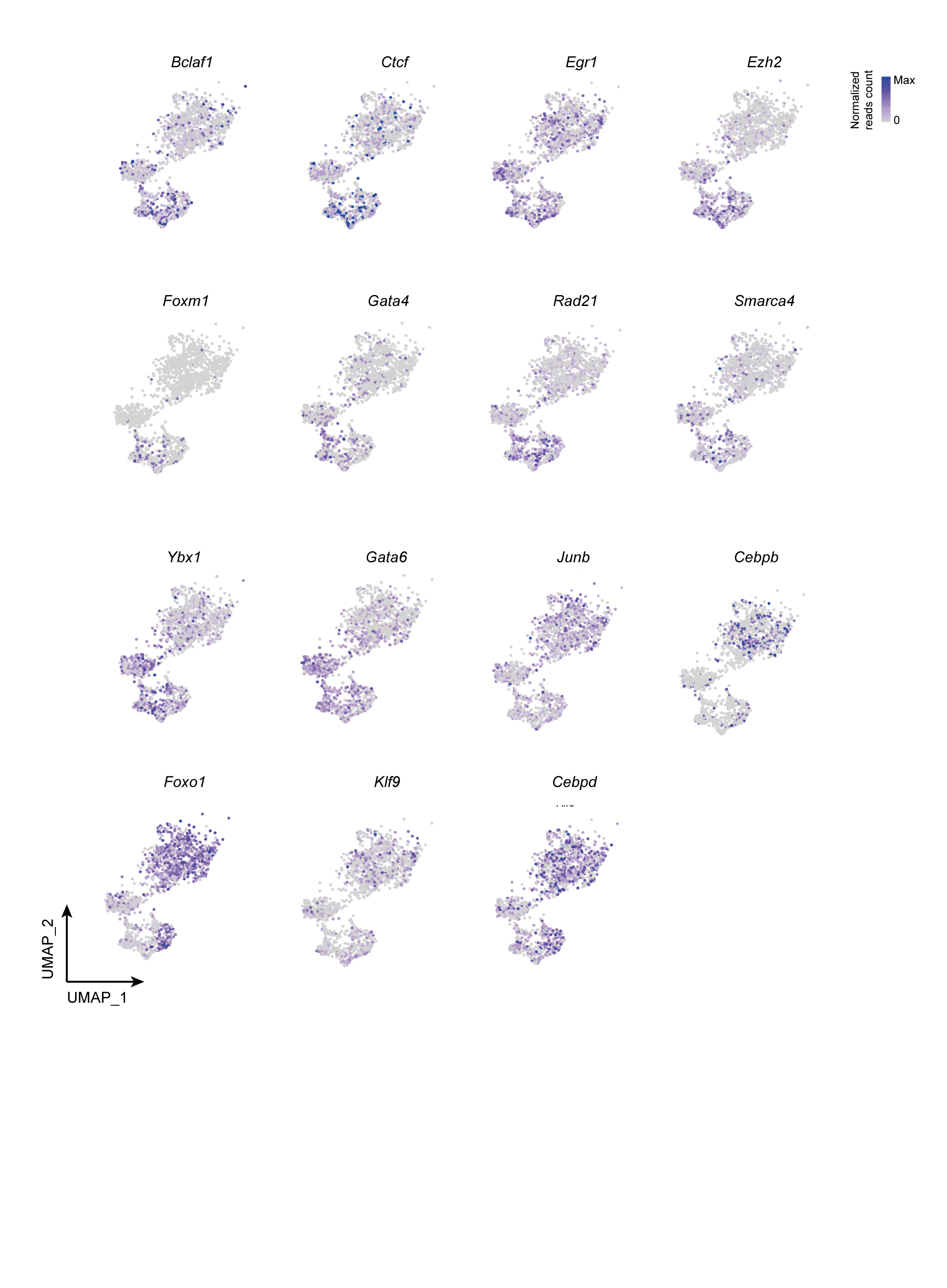


**FIGURE S4 Expression of transcriptional factors in cumulus compartments.** UMAP plots showing the expression levels of transcriptional factors in different cumulus subtypes.


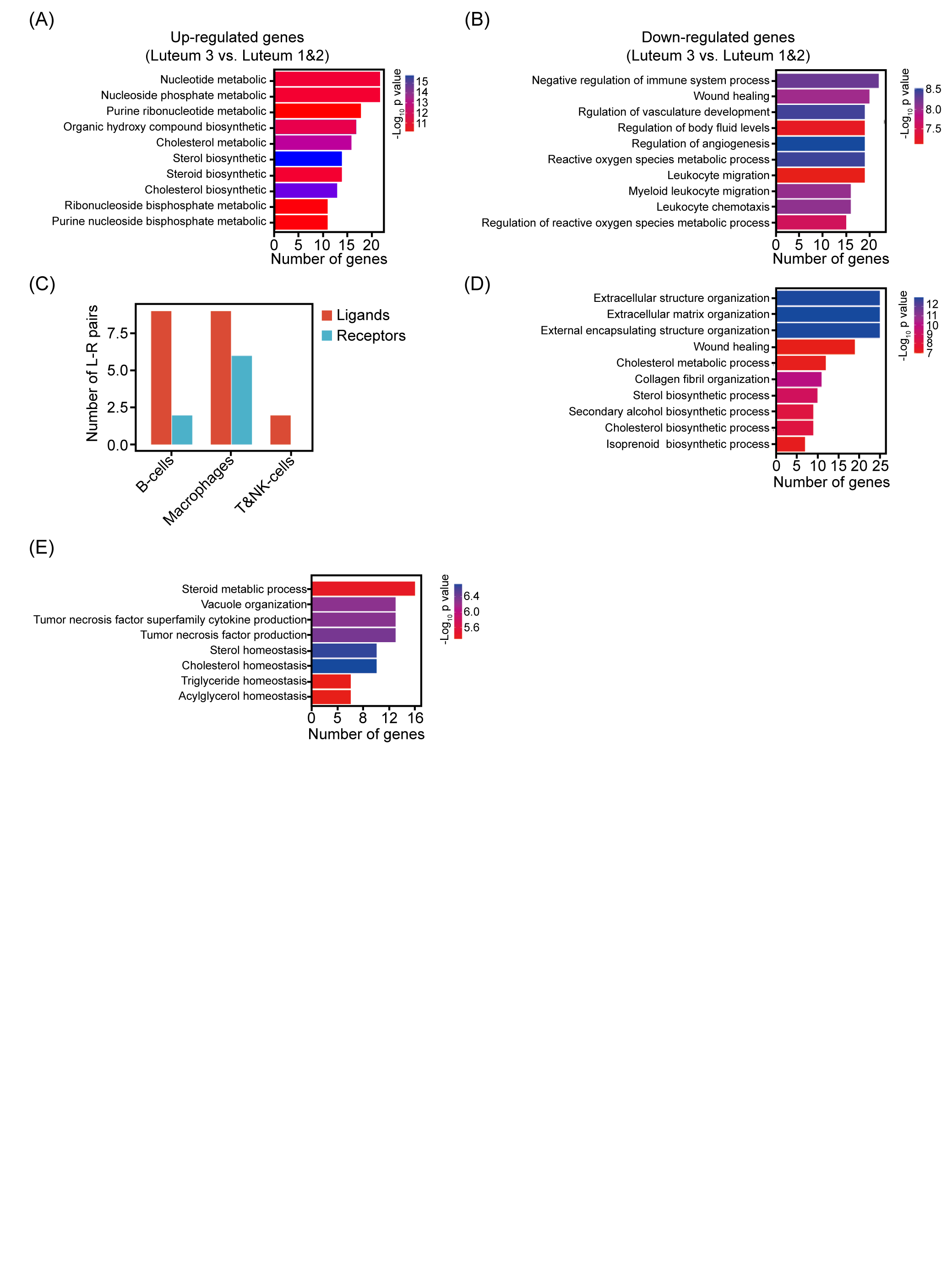


**FIGURE S5 The relationship between immune cells and luteal remodeling.** (A and B) GO terms of up- (A) and down-regulated (B) gene sets of luteum (1 and 2) and luteum 3. (C) Bar plot showing the significantly enriched L-R pairs between luteal and immune subtypes. (D and E) Top 10 significant GO terms of specifically expressed genes with gradient increased (D) or declined (E) expression levels from each individual cell to the center of the corpus luteum.
